# Molecular characterization of *Cryptosporidium* spp. and *Giardia duodenalis* from yaks in the central western region of China

**DOI:** 10.1186/s12866-015-0446-0

**Published:** 2015-05-21

**Authors:** Meng Qi, Jinzhong Cai, Rongjun Wang, Junqiang Li, Fuchun Jian, Jianying Huang, Huan Zhou, Longxian Zhang

**Affiliations:** College of Animal Science and Veterinary Medicine, Henan Agricultural University, Zhengzhou, 450002 P. R. China; International Joint Research Laboratory for Zoonotic Diseases of Henan, Zhengzhou, 450002 P. R. China; Qinghai Academy of Veterinary Medicine and Animal Science, Xining, 810016 P. R. China

**Keywords:** *Cryptosporidium*, *Giardia duodenalis*, Yaks, Genotyping, Subtyping

## Abstract

**Background:**

*Cryptosporidium* spp. and *Giardia duodenalis* are important causes of diarrheal diseases in humans and animals worldwide, and there is an increased interest in the role of animals in the mechanical transmission of these protozoa. To examine the role of yaks in this process, we examined the occurrence and genotypes of *Cryptosporidium* and *G. duodenalis* in yaks in western China*.*

**Results:**

A total of 545 fecal specimens were collected from yaks from nine different counties in the central western region of China. The prevalence for *Cryptosporidium* spp. and *G. duodenalis* were 4.0 % (22/545) and 6.0 % (16/545), respectively. Mixed infections of *Cryptosporidium* and *G. duodenalis* were also detected in four specimens. The prevalence of both protozoa differed significantly between some age groups, with higher rates of infection in animals < 1 year old. Sequence analysis of the small subunit rRNA (SSU rRNA) gene of the *Cryptosporidium* isolates identified the species as *C. parvum* (*n* = 12), *C. bovis* (*n* = 6), *C. ryanae* (*n* = 3), and *C. ubiquitum* (*n* = 1). Genotyping based on 60-kDa glycoprotein (*gp60*) gene from five *C. parvum* isolates identified all as IId with three isolates identified as IIdA15G1, one as IIdA18G1, and one as IIdA19G1. One *C. ubiquitum* isolate was identified as subtype VIIa. Amongst the *G. duodenalis* isolates, 16 were identified as assemblage E at the SSU rRNA gene. Four novel glutamate dehydrogenase (*gdh*) subtypes and two triosephosphate isomerase (*tpi*) subtypes were found amongst the *G. duodenalis* assemblage E isolates.

**Conclusions:**

The presence of *C. parvum* subtype IIdA15G1, IIdA18G1, and IIdA19G1 isolates further confirms the dominance of the *C. parvum* IId subtypes in China. These findings also indicate that yaks may be a source of zoonotic *Cryptosporidium* infection, and this is the first report of *G. duodenalis* in yaks. The data presented here provides the basis for further genotyping or subtyping studies of *G. duodenalis* in yaks.

**Electronic supplementary material:**

The online version of this article (doi:10.1186/s12866-015-0446-0) contains supplementary material, which is available to authorized users.

## Background

*Cryptosporidium* and *Giardia* are common parasitic protists that mainly cause enteric disease in humans and animals, including livestock, companion animals, and wildlife [1, 2]. Cryptosporidiosis and giardiasis result from fecal-oral transmission of oocysts or cysts, usually via water, food, or direct contact [3–5]. Livestock are often implicated in the disease cycle, and have been identified as the sources of several foodborne and waterborne outbreaks of human cryptosporidiosis and giardiasis [4–6].

To date, 26 *Cryptosporidium* species and more than 70 genotypes have been recognized [7, 8]. *Giardia duodenalis* (syn. *G. lamblia*, *G. intestinalis*) is considered a multispecies complex, with at least eight distinct genetic groups or assemblages (A–H) based on protein or DNA polymorphisms [1]. *C. hominis*, *C. parvum*, and *G. duodenalis* assemblages A and B are responsible for the majority of known human disease cases [4]. Molecular epidemiological studies conducted in various countries suggest that cattle may be a significant reservoir of *Cryptosporidium* and *G. duodenalis*, with potential for zoonotic transfer to humans [3, 4]. Cattle have been identified as the primary host for five *Cryptosporidium* species (*C. andersoni*, *C. bovis*, *C. parvum*, *C. ryanae*, and *C. ubiquitum*), and with the exception of *C. ryanae*, all of these species can also cause infection in humans [2, 9–12]. For *G. duodenalis*, assemblage E is the most commonly reported genotype in cattle, followed by assemblages A and B [13, 14].

All known species and genotypes of both *Cryptosporidium* and *G. duodenalis* have been reported worldwide in dairy and beef cattle. In contrast, very little is known about the prevalence and molecular characteristics of these pathogens in other members of the family Bovidae, including yaks (*Bos grunniens*). There has been wide variation in the reported prevalence of *Cryptosporidium* in fecal specimens from yaks in China using microscopy, enzyme immunoassays, and molecular tools for identification (5.26–39.7 %) [8, 15–24]. To date, six *Cryptosporidium* species (*C. andersoni*, *C. bovis*, *C. parvum*, *C. ryanae*, *C. ubiquitum*, and *C. xiaoi*) and three genotypes, which have been identified in yaks [8, 17, 18, 20, 24]. However, *G. duodenalis* has not been reported in yaks. The objectives of the present study were to identify the species and/or genotypes of *Cryptosporidium* and *G. duodenalis* infecting yaks in western China, and to clarify their public health significance.

## Results

### The prevalence of *Cryptosporidium* spp.

**Fig. 1:**
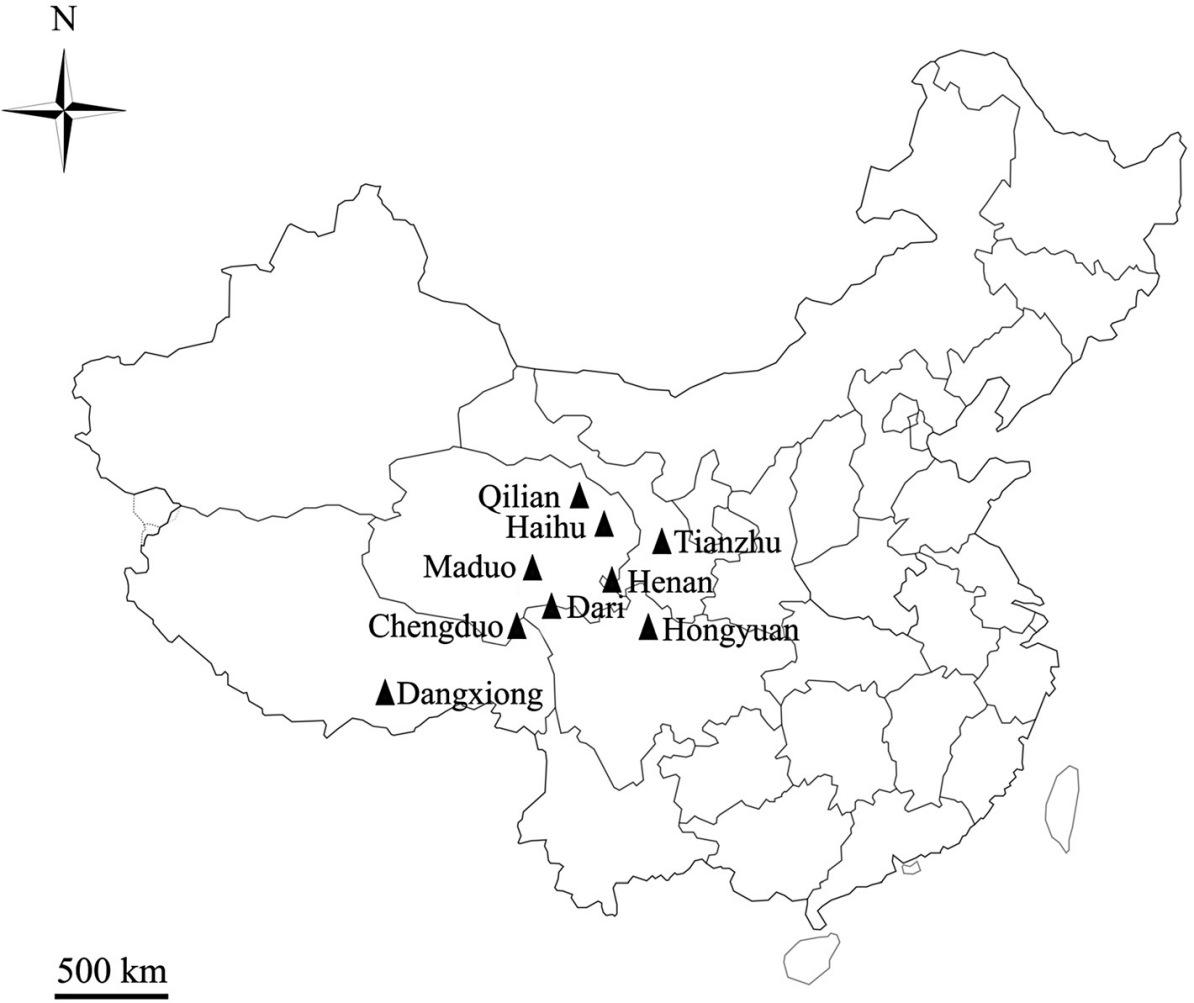
Specific locations at which specimens were collected in this study. ▲ study locations

We have collected a total number of 545 fresh yak fecal samples from 9 locations in the central western region of China between 2009–2012 to study the prevalence of *Cryptosporidium* spp. and *G. duodenalis* by PCR and sequence analysis (Fig. 1). Among them, 22 specimens were *Cryptosporidium*-positive by PCR amplification of the SSU rRNA gene, with an overall prevalence of 4.0 % (22/545). The prevalence of *Cryptosporidium* from animals at the different collection sites ranged from 0–11.8 %, with the highest prevalence in Henan County (Table 1). The most common species was *C. parvum* (12 specimens), followed by *C. bovis* (6 specimens) (Table 1). Results of the *χ*^2^ test showed that the differences in *Cryptosporidium* prevalence was not statistically significant between sampling sites (*p* > 0.05, *χ*^2^ = 14.17). However, the prevalence were significantly different between different age groups (*p* < 0.01, *χ*^2^ = 8.42), with the higher prevalence (6.6 %) observed in animals < 1 year old than animals > 1 year old (1.5 %).

**Table 1:** Prevalence and species/subtype distributions of Cryptosporidium spp. in yaks

Location	No. of yaks	No. positive (%)	Age			
			<1 year		>1 year	
			No. positive/No. of yaks	Species (n)/subtype (n)	No. positive/No. of yaks	Species (n)/subtype (n)
Tianzhu	117	7 (6.0, 4.1–7.9 CI^a^)	5/40	*C. bovis* (1), *C. ryanae* (2), *C. parvum* (2)/IIdA15G1 (2)	2/77	*C. bovis* (1), *C. ubiquitum* (1)/XIIa (1)
Dangxiong	44	4 (9.1, 4.7–13.8 CI)	4/44	*C. parvum* (4)/IIdA19G1 (1)		
Hongyuan	84	1 (1.2, 0–2.9 CI)	1/36	*C. parvum* (1)	0/48	
Henan	34	4 (11.8, 6.2–17.4 CI)	3/21	*C. bovis* (1),	1/13	*C. bovis* (1)
				*C. parvum* (2)/IIdA15G1 (1)		
Dari	62	2 (3.2, 0.5– 5.9 CI)	2/30	*C. bovis* (1),	0/32	
				*C. parvum* (2)/IIdA18G1 (1)		
Haihu	66	2 (3.0, 0.5–5.5 CI)	1/34	*C. parvum* (1)	1/32	*C. parvum* (1)
Maduo	39	1 (2.6, 0–6.2 CI)	1/20	*C. ryanae* (1)	0/19	
Qilian	47	1 (2.1, 0–5.1 CI)	1/25	*C. bovis* (1)	0/22	
Chengduo	52	0	0/24		0/28	
Total	545	22 (4.0, 3.5–4.5 CI)	18/274 (6.6)^b^	*C. bovis* (4), *C. ryanae* (3), *C. parvum* (11)/IIdA15G1 (3), IIdA18G1 (1), IIdA19G1 (1)	4/271 (1.5)^c^	*C. bovis* (2)*, C. parvum* (1), *C. ubiquitum* (1)/XIIa (1)

^a^ CI: 95 % confidence intervals

^b^ and ^c^ have significant difference

Subtyping analysis at the *gp60* gene was successful for five of the 12 *C. parvum* isolates, and all were identified as belonging to family IId: three were IIdA15G1, one was IIdA18G1, and one was IIdA19G1 (Table 1). One *C. ubiquitum* isolate was subtyped as family XIIa [GenBank: KP334140].

Mixed infections of both *Cryptosporidium* and *G. duodenalis* were also detected in four specimens: two from Tianzhu County, one from Henan County, and one from Dari County.

### The prevalence of *G. duodenalis*

A total of 16 specimens showed positive amplification of the SSU rRNA gene, all belonging to the assemblage E. The overall prevalence for *G. duodenalis* carriage was 2.9 % (16/545). The nucleotide sequences were identical to a reference sequence from a dairy cattle isolate in China [GenBank: KF843921]. The prevalence of *G. duodenalis* at the different collected sites ranged from 0–5.9 %, with the highest prevalence in Henan County (Table 2). The prevalence was not statistically significant at different sampling sites (*p* > 0.05, *χ*^2^ = 5.06); however, significant difference was observed between the prevalence in different age groups (*p* < 0.01, *χ*^2^ = 8.62), with the higher prevalence (5.1 %) observed in animals < 1 year old than animals > 1 year old (0.7 %).

**Table 2:** Prevalence and assemblages of Giardia duodenalis in yaks

Location	No. of yaks	No. positive (%)	Age			
			<1 year		>1 year	
			No. positive/No. of yaks	Assemblage (n)	No. positive/No. of yaks	Assemblage (n)
Tianzhu	117	4 (3.4, 1.7–5.1 CI^a^)	3/40	E (3)	1/77	E (1)
Dangxiong	44	0	0/44			
Hongyuan	84	1 (1.2, 0–2.9 CI)	1/36	E (1)	0/48	
Henan	34	2 (5.9, 1.1–10.7 CI)	2/21	E (2)	0/13	
Dari	62	3 (4.8, 1.7–7.7 CI)	3/30	E (3)	0/32	
Haihu	66	3 (4.5, 1.7–7.3 CI)	3/34	E (3)	0/32	
Maduo	39	1 (2.6, 0–6.2 CI)	0/20		1/19	E (1)
Qilian	47	1 (2.1, 0–5.1 CI)	1/25	E (1)	0/22	
Chengduo	52	1 (1.9, 0–4.6 CI)	1/24	E (1)	0/28	
Total	545	16 (2.9, 2.4–3.4 CI)	14/274 (5.1)^b^	E (14)	2/271 (0.7)^c^	E (2)

^a^ CI: 95 % confidence intervals

^b^ and ^c^ have significant difference

The genetic diversity of the *G. duodenalis* assemblage E isolates was determined by amplification and sequencing of the *tpi*, *gdh*, and β-giardin (*bg*) genes, with 11 *tpi*, six *gdh*, and three *bg* gene sequences obtained (Table 3). Subtype E1 (*n* = 7) was the most common subtype at the *tpi* gene. At the *gdh* gene, four subtypes of assemblage E sequences have not been reported previously.

**Table 3:** Assemblages of Giardia duodenalis determined by sequence analysis of the SSU rRNA, gdh, tpi, and bg genes of each positive specimen

Yaks ID	Location	Age	SSU rRNA	*tpi*	*gdh*	*bg*
M2	Tianzhu	3 months	E	E1		
M22	Tianzhu	3 months	E	E2		
M30	Tianzhu	4 months	E			
M69	Tianzhu	3 years	E			
607	Henan	6 months	E	E3	E1	E1
615	Henan	6 months	E	E3		E1
702	Dari	7 months	E	E3	E2	
708	Dari	8 months	E		E2	
711	Dari	8 months	E	E3	E3	E1
768	Haihu	6 months	E			
772	Haihu	6 months	E	E4	E4	
777	Haihu	6 months	E	E4	E5	
947	Maduo	2 years	E	E3		
978	Qilian	8 months	E	E3		
1045	Chengduo	6 months	E	E3		
1148	Hongyuan	3 months	E			

**Fig. 2:**
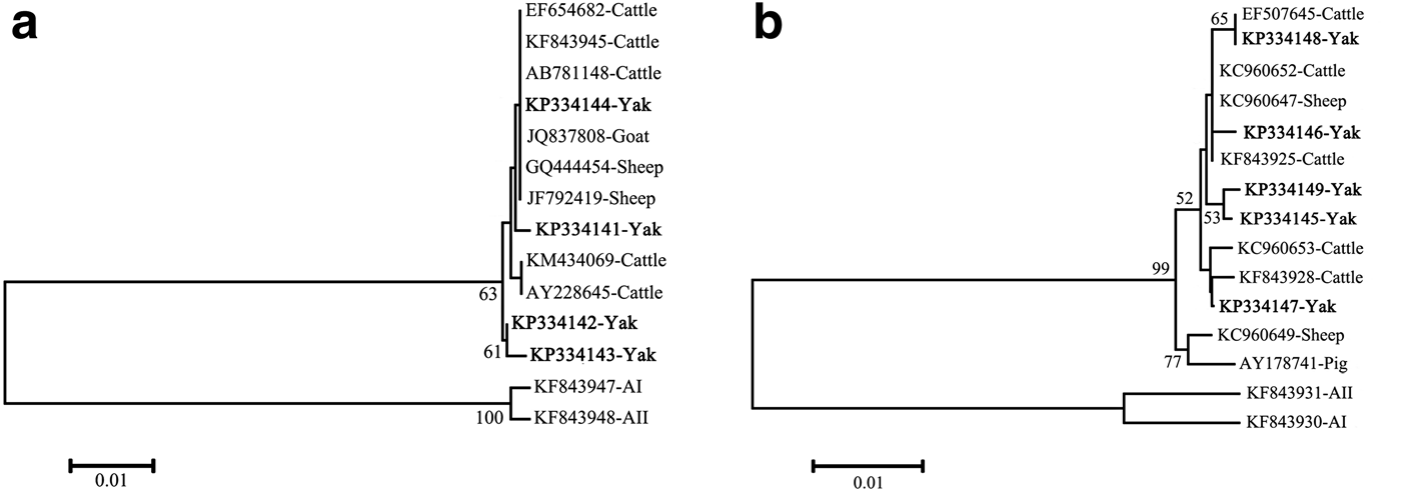
Dendrograms of *Giardia duodenalis* based on nucleotide sequences of the triosephosphate isomerase (*tpi*) (**a**) and glutamate dehydrogenase (*gdh*) (**b**) gene. Trees were constructed using the neighbor-joining method based on genetic distance calculated by the Kimura 2-parameter model, implemented in MEGA version 5.2. Bootstrap values > 50 % from 1,000 replicates are shown on nodes. Sequences from this study are marked by filled triangles

To clarify the genetic relationships between the different subtypes, alignment and phylogenetic analysis of the obtained *tpi* and *gdh* sequences with reference sequences were performed. The phylogenetic analysis of *tpi* sequences in this study with the reference subtypes AI, AII, subtypes E from cattle, goat and sheep demonstrated that subtypes E1 [GenBank: KP334141] and E4 [GenBank: KP334144] clustered with reference subtypes E, whereas subtypes E2 [GenBank: KP334142] and E3 [GenBank: KP334143] formed one separate cluster in assemblage E (Fig. 2a). Alignment and phylogenetic analysis of the obtained *gdh* sequences with reference sequences indicated the presence of only *G. duodenalis* subtype E from cattle and sheep, although the genetic variation was noticed within this subtype (Fig. 2b).

## Discussion

In the present study, the prevalence of *Cryptosporidium* and *G. duodenalis* in yaks in western China was 4.0 % and 2.9 %, respectively. To the best of our knowledge, this is the first report of *G. duodenalis* in yaks. The prevalence of *Cryptosporidium* species appears to vary widely depending on the geographic area in China. The overall *Cryptosporidium* prevalence in this study was similar to that reported by Qin et al. (5.26 %) [24], but was lower than the majority of reported rates (10.4–39.7 %) [8, 15–23]. Infection rates are affected by many factors, including animal age, specimen size, diagnostic tests, management systems, seasons, and geographic area. Therefore, it is difficult to compare prevalence data between studies. However, similar to our results, previous studies have shown that weaned calves and yearlings are more frequently infected with *Cryptosporidium* than older yaks [8, 20]. The overall *G. duodenalis* infection rate in this study is lower than rates recently reported in diary cattle in Heilongjiang Province (5.2 %, 41/814) and Henan Province (7.2 %, 128/1777), China [25, 26]. We also determined that there was a statistically significant difference in *G. duodenalis* infection rates between the different age groups. This finding was similar to previous reports regarding giardiasis as a common infection in immature animals [25–27].

To date, in addition to the four most common *Cryptosporidium* species (*C. parvum*, *C. andersoni*, *C. bovis*, and *C. ryanae*), *C. ubiquitum* and *C. xiaoi* have also been identified in a small number of yaks [8, 20, 24]. A previous study of *Cryptosporidium* in yaks in Qinghai Province reported that *C. bovis* was the most common species (31/55, 56.4 %), followed by *C. parvum* (16/55, 29.1 %), and *C. ryanae* (5/55, 9.0 %) [20]. However, another study of *Cryptosporidium* in yaks in the same area reported that *C. bovis* (56/98, 57.1 %) and *C. ryanae* (33/98, 33.7 %) were the most common species [8], followed by *C. andersoni* (2/98, 2.0 %), *C. ubiquitum* (1/98, 1.0 %), and *C. xiaoi* (1/98, 1.0 %), and that *C. parvum* was not detected. The results of these previous studies suggest that there is some variation in the dominant species causing cryptosporidiosis in yaks in Qinghai Province. Of the four *Cryptosporidium*-positive specimens in Tianzhu County, three were identified as *C. bovis* and one was identified as *C. andersoni*, which is similar to a previous study showing that *C. bovis* is most prevalent in yaks [24]. In the present study, four *Cryptosporidium* species were identified in yaks, with the most abundant species being *C. parvum* (12/22, 54.5 %). This result differs from previous reported in yaks, whereas agrees with most previous reports in dairy calves found that *C. parvum* was the most common species, especially in preweaned dairy calves in the Ningxia Hui Autonomous Region, northwestern China [28–31].

Of the four species identified, *C. parvum* is a major pathogen in humans, while *C. ubiquitum* has been identified in many human cases of cryptosporidiosis in the United Kingdom, Slovenia, the United States, Canada, Spain, and New Zealand [3, 32]. Of the 22 *Cryptosporidium*-positive isolates typed at the SSU rRNA gene, 12 were *C. parvum* and one was *C. ubiquitum*. The *C. ubiquitum* isolate belonged to the family XIIa subtype of *gp60*, which has been detected in goats in China, as well as in humans and other animals in multiple countries [32–34]. Five of the *C. parvum* isolates were identified as belonging to the IId subtype, and differ from the IIa subtype isolates found in yaks in Qinghai Province [8]. Generally, of the 14 *C. parvum* subtypes (IIa–IIi, and IIk–IIo), IIa and IId are most commonly associated with zoonosis, while subtypes IIc and IIe are anthroponotic subtype families [35]. In general, the *C. parvum* subtypes found in China appear to be unique. While Mi et al. [8, 34] reported *C. parvum* subtype IIa isolates in yaks and goats, Ye et al. [36] reported subtype IIc isolate in monkeys, all other *C. parvum* isolates from China have belonged to IId subtypes, including IIdA15G1 in rodents and cattle [28, 29], and IIdA19G1 in cattle, humans, goats, and urban wastewater [30, 31, 34, 37, 38]. Subtype IIdA18G1 has previously been reported in calves in Serbia and Montenegro [39], in lambs in Spain [40], and in humans in Kuwait and the United Kingdom [41, 42]. In the present study, *C. parvum* subtype IIdA15G1 was the predominant subtype in yaks in western China, which further confirms the dominance of the *C. parvum* IIdA15G1 subtype in western China.

Numerous molecular epidemiological data have shown that most cattle, sheep, and pigs are infected with the host-specific *G. duodenalis* assemblage E (livestock genotype) [1]. Although *G. duodenalis* assemblage A and B isolates have been detected in cattle in China, most test-positive specimens have been confirmed as assemblage E [25, 26, 29]. A recent study reported that *G. duodenalis* assemblage E may cause intestinal lesions, leading to calf scours [43]. In the present study, sequence analyses indicated that the yaks were also infected with livestock-specific *G. duodenalis* assemblage E. The previous study in Heilongjiang and Henan Provinces, China, found that the *G. duodenalis* assemblage E subtypes in cattle may represent the endemic genetic characteristics [25, 26, 44]. Similar to previous reports, the current study also identified a high level of genetic polymorphism within assemblage E isolates in yaks by subtyping *tpi* and *gdh* sequences. More data from large-scale molecular epidemiological investigations focusing in assemblage E strains will clarify the geographical distribution of this pathogen.

## Conclusion

*Cryptosporidium parvum* is the predominant species in yaks in the study area, which differs from previous reports in Qinghai Provinces that identified *C. bovis* as the most common species. The presence of *C. parvum* subtype IIdA15G1, IIdA18G1, and IIdA19G1 isolates further confirms the dominance of the *C. parvum* IId subtypes in China. These findings indicate that yaks may be a source of zoonotic *Cryptosporidium*. This is the first report of *G. duodenalis* in yaks, and the obtained data provide useful information for further genotyping or subtyping studies of *G. duodenalis*. More studies are required to determine the dramatic geographic differences in the prevalence of zoonotic *Cryptosporidium* and *G. duodenalis* in bovine animals in China.

## Methods

### Ethics statement

This study was performed according to the recommendations of the Guide for the Care and Use of Laboratory Animals of the Ministry of Health, China. The research protocol was reviewed and approved by the Research Ethics Committee of Henan Agricultural University. Permission was obtained from the animals’ owners prior to the collection of fecal specimens.

### Study area and specimen collection

From August 2009 to September 2012, fresh fecal specimens were collected from yaks from 9 locations in the central western region of China (Fig. 1). Collection sites included: Henan, Dari, Haihu, Maduo, Qilian, and Chengduo counties in Qinghai Province (89°35′E–103°04′E, 31°90′N–39°19′N); Tianzhu county in Gansu Province (102°07′E–103°46′E, 36°31′N–37°55′N); Dangxiong county in the Xizang Tibetan Autonomous Region (90°45′E–91°31′E, 29°31′N–31°04′N); Hongyuan county in Sichuan Province (101°51′E–103°23′E, 31°50′N–33°22′N). Yaks in these areas were kept outdoors and shared pastures with sheep and wild animals. Fresh feces were collected from the ground if the animal was observed to defecate, with care taken to avoid environmental contamination by sampling only those portions of the fecal material that had not been in contact with the ground. A total of 545 fresh fecal specimens were collected immediately post-defecation from yaks aged 2 months to 10 years. No obvious clinical signs were observed in the animals. All specimens were stored in 2.5 % (w/v) potassium dichromate solution at 4 °C until DNA extraction.

### DNA extraction

Specimens were washed three times in distilled water with centrifugation at 3000 × *g* for 10 min to remove the potassium dichromate. DNA was extracted from 200 mg of each fecal specimen using the E.Z.N.A.R Stool DNA Kit (Omega Biotek Inc., Norcross, USA) according to the manufacturer’s instruction. The extracted DNA was stored at −20 °C.

### PCR amplification

*Cryptosporidium* species were detected in the fecal specimens and differentiated by PCR analysis of the small subunit rRNA (SSU rRNA) gene according to previous process [45]. *C. parvum* and *C. ubiquitum* were subtyped based on sequence analysis of the 60-kDa glycoprotein gene (*gp60*) following PCR amplification [32, 46].

*G. duodenalis* genotyping was performed using nested PCR amplification of the the SSU rRNA region from each specimen as described previously [47]. DNA from all SSU rRNA-positive specimens were subjected to further PCR analysis to detect the presence of the glutamate dehydrogenase (*gdh*), triose phosphate isomerase (*tpi*), and β-giardin (*bg*) genes based on previously described methods [48–50].

The primers used in the PCR analysis of all gene targets, the annealing temperatures, and the sizes of the expected PCR products according to previous described. The PCR reactions for all genes were conducted in 25 μL reaction mixtures containing of 1 × PCR buffer (TaKaRa Shuzo Co., Ltd., Otsu, Japan), 200 μM each dNTP, 0.4 μM each primer, 1 unit of TaKaRa rTaq DNA polymerase, and 2 μL of DNA. Except the SSU rRNA protocol, 1 × GC buffer II and LA Taq DNA polymerase (TaKaRa Shuzo Co., Ltd.) were used instead of 1 × PCR buffer and rTaq. The secondary PCR products were examined by agarose gel electrophoresis and visualised after GelRed™ (Biotium Inc., Hayward, CA, USA) staining.

### Sequence analysis

All PCR amplicons were sequenced on an ABI PRISM 3730 XL DNA Analyzer using a BigDye Terminator v3.1 Cycle Sequencing Kit (Applied Biosystems, Foster City, CA, USA). The sequence accuracy was confirmed by two-directional sequencing, and sequences were identified by alignment with reference sequences downloaded from GenBank (http://www.ncbi.nlm.nih.gov) using MEGA 5 software (http://www.megasoftware.net/). The subtypes of *G. duodenalis* identified at the *tpi* and *gdh* genes in this study were compared with known ones using a neighbour-joining analysis of the aligned sequences using MEGA 5 software (http://www.megasoftware.net/). The reliability of these trees was assessed by the bootstrap analysis with 1000 replicates, with the substitution type of nucleotide. The nucleotide sequences obtained in this study have been deposited in GenBank under accession numbers [GenBank: KP334133-KP334150] (Additional file 1).

### Statistical analysis

The *χ*^2^ test was used to compare the *Cryptosporidium* and *G. duodenalis* infection rates, and differences were considered significant when *p* < 0.05.

## Additional file

Additional file 1:
**GenBank accession numbers and nucleotide sequences in this study.**
