# Supplementary material for: Molecular characterization of Cryptosporidium spp. and Giardia duodenalis from yaks in the central western region of China
Source: BMC Microbiol. 2015 May 21;15:108. doi: 10.1186/s12866-015-0446-0 (PMC4438589; doi:10.1186/s12866-015-0446-0)
Supplement: Additional file 1: — GenBank accession numbers and nucleotide sequences in this study. [file 12866_2015_446_MOESM1_ESM.doc]

**Availability of SupportingData**

Thank you for your direct submission of sequence data to GenBank. We have provided GenBank accession numbers for your nucleotide sequences:

Yaksequence2.sqn M3ryanae KP334133

Yaksequence2.sqn M10ubiquitum KP334134

Yaksequence2.sqn 702bovis KP334135

Yaksequence2.sqn 704parvum KP334136

Yaksequence2.sqn 615IIdA15G1 KP334137

Yaksequence2.sqn 704IIdA18G1 KP334138

Yaksequence2.sqn 8IIdA19G1 KP334139

Yaksequence2.sqn M10XIIa KP334140

Yaksequence2.sqn 2E1TPI KP334141

Yaksequence2.sqn 22E2TPI KP334142

Yaksequence2.sqn 615E3TPI KP334143

Yaksequence2.sqn 772E4TPI KP334144

Yaksequence2.sqn 607E1GDH KP334145

Yaksequence2.sqn 702E2GDH KP334146

Yaksequence2.sqn 711E3GDH KP334147

Yaksequence2.sqn 772E4GDH KP334148

Yaksequence2.sqn 777E5GDH KP334149

Yaksequence2.sqn 711assemblageE KP334150

>KP334133

TTAGATAAAGAACCAATATTTTTGGTGACTCATAATAACTTTACGGATCACACTATGTGACATATCATTCAAGTTTCTGACCTATCAGCTTTAGACGGTAGGGTATTGGCCTACCGTGGCTATGACGGGTAACGGGGAATTAGGGTTCGATTCCGGAGAGGGAGCCTGAGAAACGGCTACCACATCTAAGGAAGGCAGCAGGCGCGCAAATTACCCAATCCTAATACAGGGAGGTAGTGACAAGAAATAACAATACAGAGCCTTACGGTTTTGTAATTGGAATGAGTTAAGTATAAACCCCTTAACAAGTATCAATTGGAGGGCAAGTCTGGTGCCAGCAGCCGCGGTAATTCCAGCTCCAATAGCGTATATTAAAGTTGTTGCAGTTAAAAAGCTCGTAGTTAATTTTCTGTTAATTTTTATATACAATGCTACGGTATTTATATAATATTAACATAATTCATATTACTTTTTAGTATATGAAACTTTACTTTGAGAAAATTAGAGTGCTTAAAGCAGGCTATTGCCTTGAATACTCCAGCATGGAATAATATTAAGGATTTTTATTCTTCTTATTGGTTCTAGAATAAAAATAATGATTAATAGGGACAGTTGGGGGCATTTGTATTTAACAGTCAGAGGTGAAATTCTTAGATTTGTTAAAGACAAACTACTGCGAAAGCATTTGCCAAGGATGTTTTCATTAATCAAGAACGAAAGTTAGGGGATCGAAGACGATCAGATACCGTCGTAGTCTTAACCATAAACTATGCCAACTAGAGATTGGAGGT

>KP334134

GGGTTGTATTTATTAGATAAAGAACCAATTTAATTTGGTGATTCATAATAACTTTACGGATCACATTTATATGTGACATATCATTCAAGTTTCTGACCTATCAGCTTTAGACGGTAGGGTATTGGCCTACCGTGGCAATGACGGGTAACGGGGAATTAGGGTTCGATTCCGGAGAGGGAGCCTGAGAAACGGCTACCACATCTAAGGAAGGCAGCAGGCGCGCAAATTACCCAATCCTAATACAGGGAGGTAGTGACAAGAAATAACAATACAGGACTTTAAATAGTTTTGTAATTGGAATGAGTTAAGTATAAACCCCTTTACAAGTATCAATTGGAGGGCAAGTCTGGTGCCAGCAGCCGCGGTAATTCCAGCTCCAATAGCGTATATTAAAGTTGTTGCAGTTAAAAAGCTCGTAGTTGGATTTCTGTTAATAATTTATATATAATATTTTATTAATATTTATATAGTATTAACATAATTCATATTACTATATTTTATAGTATATGAAATTTTACTTTGAGAAAATTAGAGTGCTTAAAGCAGGCATTAGCCTTGAATACTCCAGCATGGAATAATATAAAAGATTTTTATCTTTTTTATTGGTTCTAAGATAAAAATAATGATTAATAGGGACAGTTGGGGGCATTTGTATTTAACAGTCAGAGGTGAAATTCTTAGATTTGTTAAAGACAAACTAGTGCGAAAGCATTTGCCAAGGATGTTTTCATTAATCAAGAACGAAAGTTAGGGGATCGAAGACGATCAGATACCGTCGTAGTCTTAACCATAAACTATGCCAACTAGAGATTGGAGGTTGTTCCTTACTCC

>KP334135

TTATTAGATAAAGAACCAATATTTTTGGTGACTCATAATAACTTTACGGATCACATTATGTGACATATCATTCAAGTTTCTGACCTATCAGCTTTAGACGGTAGGGTATTGGCCTACCGTGGCTATGACGGGTAACGGGGAATTAGGGTTCGATTCCGGAGAGGGAGCCTGAGAAACGGCTACCACATCTAAGGAAGGCAGCAGGCGCGCAAATTACCCAATCCTAATACAGGGAGGTAGTGACAAGAAATAACAATACAGAGCCTTACGGTTTTGTAATTGGAATGAGTTAAGTATAAACCCCTTAACAAGTATCAATTGGAGGGCAAGTCTGGTGCCAGCAGCCGCGGTAATTCCAGCTCCAATAGCGTATATTAAAGTTGTTGCAGTTAAAAAGCTCGTAGTTAATTTTCTGTTAATTTTTATATACAATGCTACGGTATTTATATAATATTAACATAATTCATATTACTTTTTAGTATATGAAACTTTACTTTGAGAAAATTAGAGTGCTTAAAGCAGGCTATTGCCTTGAATACTCCAGCATGGAATAATATTAAGGATTTTTATTCTTCTTATTGGTTCTAGAATAAAAATAATGATTAATAGGGACAGTTGGGGGCATTTGTATTTAACAGTCAGAGGTGAAATTCTTAGATTTGTTAAAGACAAACTACTGCGAAAGCATTTGCCA

AGGATGTTTTCATTAATCAAGAACGAAAGTTAGGGGATCGAAGACGATCAGATACCGTCGTAGTCTTAACCATAAACTATGCCAACTAGAGATTGGAGGTTGTTCCTTACTCCTT

>KP334136

AACCAATATAATTGGTGACTCATAATAACTTTACGGATCACATTAAATGTGACATATCATTCAAGTTTCTGACCTATCAGCTTTAGACGGTAGGGTATTGGCCTACCGTGGCAATGACGGGTAACGGGGAATTAGGGTTCGATTCCGGAGAGGGAGCCTGAGAAACGGCTACCACATCTAAGGAAGGCAGCAGGCGCGCAAATTACCCAATCCTAATACAGGGAGGTAGTGACAAGAAATAACAATACAGGACTTTTTGGTTTTGTAATTGGAATGAGTTAAGTATAAACCCCTTTACAAGTATCAATTGGAGGGCAAGTCTGGTGCCAGCAGCCGCGGTAATTCCAGCTCCAATAGCGTATATTAAAGTTGTTGCAGTTAAAAAGCTCGTAGTTGGATTTCTGTTAATAATTTATATAAAATATTTTGATGAATATTTATATAATATTAACATAATTCATATTACTATATATTTTAGTATATGAAATTTTACTTTGAGAAAATTAGAGTGCTTAAAGCAGGCATATGCCTTGAATACTCCAGCATGGAATAATATTAAAGATTTTTATCTTTCTTATTGGTTCTAAGATAAGAATAATGATTAATAGGGACAGTTGGGGGCATTTGTATTTAACAGTCAGAGGTGAAATTCTTAGATTTGTTAAAGACAAACTAATGCGAAAGCATTTGCCAAGGATGTTTTCATTAATCAAGAACGAAAGTTAGGGGATCGAAGACGATCAGATACCGTCGTAGTCTTAACCATAAACTATGCCAACTAGAGATTGGAGGTTGTTCCTTACTCCTTC

>KP334137

CCCAGCCGTTCCACTCAGAGGCACTTTAAAGGATGTTTCTGTTGAGGGTTCATCATCATCATCATCATCATCATCATCGTCATCATCATCATCATCAACATCGACTGTAGCACCAACTCCAAAGAAAGAAAGAACTGGAGAGGAAGTAGGTAATCCAGGTTCTGAAGGTCAGGACGGTAAAGGAGACACTGAAGAAACAGAAGACAATCAGACCGAGAGTACTGTTTCTCAAAATACTCCAGCTCAAACTGAAGGCACAACTACCGAAACCACAGAAGCTGCTCCAAAGAAAGAGTGCGGTACTTCATTTGTTATGTGGTTCGGAGAGGGTGTTCCAGTTGCATCTTTGAAGTGTGGCGACTATACTATGGTCTATGCACCAGAAAAGGACAAAACAGATCCCGCACCAAGATATATCTCTGGTGAAGTTACATCTGTAACCTTTGAAAAACAAGAGAGCACAGTTACAATCAAGGTTAATAATGTAGAGTTCAGCACTCTTTCTACTAGCTCAAGTAGTCCAACTGAAAATAGCGGATCTGCAGGTCAGGTTCCATCAAGATCAAGAAGATCACTCTCAGAGGAGGCTAGTGAAACTGCAACCGTCGATTTGTTTGCCTTCACCCTTGATGGTGGTAAAAGAATTGAAGTTGCTGTACCAAGCGACGAAGATGCATCTAAAAGAAACCAGTACAGTTTGGTTGCAGACGATAAACCTTTCTATACCGGCTCAAATAGCGGCGCCACTGATGGTATCTTCAGGTTGAATGAGGACGGAGACTTGGTTGACAAGGACAACAAAGTTCTTTTGAAGGAT

>KP334138

ATCGGTATAGTCTCCGCTGTATTATCAGCCCCAGCCGTTCCACTCAGAGGCACTTTAAAGGATGTTTCTGTTGAGGGTTCATCATCATCATCATCATCATCATCATCATCATCATCGTCATCATCATCATCATCAACATCGACTGTAGCACCAACTCCAAAGAAAGAAAGAACTGGAGAGGAAGTAGGTAATCCAGGTTCTGAAGGTCAGGACGGTAAAGGAGACACTGAAGAAACAGAAGACAATCAGACCGAGAGTACTGTTTCTCAAAATACTCCAGCTCAAACTGAAGGCACAACTACCGAAACCACAGAAGCTGCTCCAAAGAAAGAGTGCGGTACTTCATTTGTTATGTGGTTCGGAGAGGGTGTTCCAGTTGCATCTTTGAAGTGTGGCGACTATACTATGGTCTATGCACCAGAAAAGGACAAAACAGATCCCGCACCAAGATATATCTCTGGTGAAGTTACATCTGTAACCTTTGAAAAACAAGAGAGCACAGTTACAATCAAGGTTAATAATGTAGAGTTCAGCACTCTTTCTACTAGCTCAAGTAGTCCAACTGAAAATAGCGGATCTGCAGGTCAGGTTCCATCAAGATCAAGAAGATCACTCTCAGAGGAGGCTAGTGAAACTGCAACCGTCGATTTGTTTGCCTTCACCCTTGATGGTGGTAAAAGAATTGAAGTTGCTGTACCAAGCGACGAAGATGCATCTAAAAGAAACCAGTACAGTTTGGTTGCAGACGATAAACCTTTCTATACCGGCTCAAATAGCGGCGCCACTGATGGTATCTTCAGGTTGAATGAGGACGGAGACTTGGTTGACAAGGACAACAAA

GTACTT

>KP334139

CTCAGCCCCAGCCGTTCCACTCAGAGGCACTTTAAAGGATGTTTCTGTTGAGGGTTCATCATCATCATCATCATCATCATCATCATCATCATCATCGTCATCATCATCATCATCAACATCGACTGTAGCACCAACTCCAAAGAAAGAAAGAACTGGAGAGGAAGTAGGTAATCCAGGTTCTGAAGGTCAGGACGGTAAAGGAGACACTGAAGAAACAGAAGACAATCAGACCGAGAGTACTGTTTCTCAAAATACTCCAGCTCAAACTGAAGGCACAACTACCGAAACCACAGAAGCTGCTCCAAAGAAAGAGTGCGGTACTTCATTTGTTATGTGGTTCGGAGAGGGTGTTCCAGTTGCATCTTTGAAGTGTGGCGACTATACTATGGTCTATGCACCAGAAAAGGACAAAACAGATCCCGCACCAAGATATATCTCTGGTGAAGTTACATCTGTAACCTTTGAAAAACAAGAGAGCACAGTTACAATCAAGGTTAATAATGTAGAGTTCAGCACTCTTTCTACTAGCTCAAGTAGTCCAACTGAAAATAGCGGATCTGCAGGTCAGGTTCCATCAAGATCAAGAAGATCACTCTCAGAGGAGGCTAGTGAAACTGCAACCGTCGATTTGTTTGCCTTCACCCTTGATGGTGGTAAAAGAATTGAAGTTGCTGTACCAAGCGACGAAGATGCATCTAAAAGAAACCAGTACAGTTTGGTTGCAGACGATAAACCTTTCTATACCGGCTCAAATAGCGGCGCCACTGATGGTATCTTCAGGTTGAATGAGGACGGAGACTTGGTTGA

>KP334140

TGAGATTTTTACTCGCTATCGTATCACTCTCCGTTTTCATCTCAGTTGTATTCTCAGCTCCAGGTGTTCCACTCAGAGGTACATTGAAAGAGGATGACAGTACTAACGTTAGCACAACAACAGCTGCTCCAAAGAAGATAATCGTCAGATCTACCGAGGAAGGCACAACACCTGCTCCTACTACTCCTAGTACTACTGCTTCTACTGCTGCTCCCACTACTGTTAGTACTACCGCTCCTTCTGGCGGCGGTGTTACTCCTACTAGCACTGATGGAGATGAGACTACTGACACTGGTAGTGGTACTACCGGCGAAACAGTTACAACTACTCCTGACCCAATGGAGAAATGCGGTCTTTCATTCGTCATGTGGTTTGTTAGTGGTACTCCAGTTACAACTCTCGAATGTGGACCTTACACCATGGTATATGGACCAGTTGAAGGGGAGACTAACCCTGCAGCAAGGTATGTCTCTGGGCCCGTTACTACGGTTACTTATGAAGAAAGCAGCAAGAAACTCATGATCAATGGCCAGGAATTCGCCACTCTTTCCATGGATTCATCAAAACCAACCACAGCTACAACAACACCAGCAGCTAGGTTACTTGCAGAAGGTGGCACTGCTACAGAAGCCGTCGCAATGACTGATCTATACACCTTCACCTTAAAGGGTGGTAAAGCCATTAGTGTAGGTGTGCCTGCTGTGGACGATTCAACTAAAAGAGACAAATACAGTTTATCCGCTGATAGCCAAACATTCTACACCGGTACTGCTACCAATAGTGGTACTACTAATGGAATCTTCAAATTGAACAATGATGGTGATTTAGT

>KP334141

GTAACTTCAAGTGTAACGGCTCGCTTGATTTTATCAAGAGCCATGTGGCGGCCATTGCTGCCCACAAGATCCCCGATTCCGTAGACGTTGTTGTTGCCCCTTCTGCCGTACATTTATCAACAGCTATTGCAGCAAACACGTCAAAACAGTTGAAGATAGCGGCGCAGAATGTGTACCTAGAGGGGAATGGAGCGTGGACTGGTGAGACGAGTGTTGAGATGCTTCAGGACATGGGCTTGGAGTACGTGATAATAGGGCATTCTGAAAGGCGTAGAATCATGGGGGAGACCGACGAGCAGAGTGCCAAGAAGGCTAAGCGTGCTCTAGAAAAGGATATGACGGTTATCTTTTGTGTTGGAGAGACCCTTGATGAGCGCAAGGCCAACCGCACCATGGAGGTAAACATTGCTCAGCTTGAGGCGCTCAGCAAAGAGCTCGGAGAATCTAAGCTGCTATGGAAAAAAGTCGTTATTGCTTACGAGCCCGTATGGTCCATTGGCACGGGC

>KP334142

GTAACTTCAAGTGTAACGGCTCGCTTGATTTTATCAAGAGCCATGTGGCGGCCATTGCTGCCCACAAGATCCCCGATTCCATAGACGTTGTTGTTGCCCCTTCTGCCGTACATTTATCAACAGCTATTGCAGCAAACACGTCAAAACAGTTGAAGATAGCGGCGCAGAATGTGTACCTAGAGGGGAATGGAGCGTGGACTGGTGAGACGAGTGTTGAGATGCTTCAGGACATGGGCTTGGAGTACGTGATAATAGGGCATTCTGAAAGGCGTAGAATCATGGGGGAGACCGACGAGCAGAGCGCCAAGAAGGCTAAGCGTGCTCTAGAAAAGGATATGACGGTTATCTTTTGTGTTGGAGAGACCCTTGATGAGCGCAAGGCCAACCGCACCATGGAGGTAAACATTGCTCAGCTTGAGGCGCTCAGCAAAGAGCTCGGGGAATCTAAGCTGCTATGGAAAAAAGTCGTTATTGCTTACGAGCCCGTATGGTCCATTGGCACGGGCG

>KP334143

TTAAGTGTAACGGCTCGCTTGATTTTATCAAGAGCCATGTGGCGGCCATTGCTGCCCACAAGATCCCCGATTCCATAGACGTTGTTGTTGCCCCTTCTGCCGTACATTTATCAACAGCTATTGCAGCAAACACGTCAAAACAGTTGAAGATAGCGGCGCAGAATGTGTACCTAGAGGGGAATGGAGCGTGGACTGGTGAGACGAGTGTTGAGATGCTTCAGGACATGGGCTTGGAGTACGTGATAATAGGGCATTCTGAAAGGCGTAGAATCATGGGGGAGACCGACGAGCAGAGCGCCAAGAAGGCTAAGCGTGCTCTAGAAAAGGATATGACGGTTATCTTTTGTGTTGGAGAGACCCTTGATGAGCGCAAGGCCAACCGCACCATGGAGGTAAACATTGCTCAGCTTGAGGCGCTCAGCAAAGAGCTCGGGGAATCTAAGCTGCTATGGAAAAAAGTCATTATTGCTTACGAGCCCGTATGGTCCAT

TGGCACGGGCG

>KP334144

GTAACTTCAAGTGTAACGGCTCGCTTGATTTTATCAAGAGCCATGTGGCGGCCATTGCTGCCCACAAGATCCCCGATTCCGTAGACGTTGTTGTTGCCCCTTCTGCCGTACATTTATCAACAGCTATTGCAGCAAACACGTCAAAACAGTTGAAGATAGCGGCGCAGAATGTGTACCTAGAGGGGAATGGAGCGTGGACTGGTGAGACGAGTGTTGAGATGCTTCAGGACATGGGCTTGGAGTACGTGATAATAGGGCATTCTGAAAGGCGTAGAATCATGGGGGAGACCGACGAGCAGAGTGCCAAGAAGGCTAAGCGTGCTCTAGAAAAGGATATGACGGTTATCTTTTGTGTTGGAGAGACCCTTGATGAGCGCAAGGCCAACCGCACCATGGAGGTAAACATTGCTCAGCTTGAGGCGCTCAGCAAAGAGCTCGGGGAATCTAAGCTGCTATGGAAAAAAGTCGTTATTGCTTACGAGCCCGTATGGTCCATTGGCACGGGC

>KP334145

TTCAGAGGCACGTTGGGGCTGACACTGATGTTCCTGCCGGCGACATCGGCGTCGGCGCTCGCGAGATCGGTTACTTGTACGGACAGTACAAGCGCCTGAGGAACGAGTTTACGGGCGTCCTCACGGGCAAAAACGTCAAGTGGGGCGGGTCCTTCATCAGGCCGGAGGCCACAGGCTATGGTGCTGTCTACTTCCTGGAGGAGATGTGCAAGGACAACAACACTGTAATCAGGGGCAAGAACGTCCTCCTTTCCGGCTCCGGCAACGTCGCTCAATTTGCTTGTGAGAAGCTCCTTCAGCTCGGCGCGAAGGTCCTTACCTTTTCAGACTCCAATGGAACCATTGTCGACAAGGACGGGTTTAATGAGGAAAAGCTGGACCACCTCAAGTATCTCAAGAACGAAAAGCGTGGGCGCGTTTCTGAGTTCAAGGACAAGTATCCTGGAGTCATGTACTATGAAGGCAAGAAGCCTTGGGAGTGCTTCGAGGGCCAGGTAGATTGCATCATGCCTTGCGCCAC

>KP334146

TTCAGAGGCACGTTGGGGCTGACACTGATGTTCCTGCCGGTGACATCGGCGTCGGCGCTCGCGAGATCGGTTACTTGTACGGACAGTACAAGCGCCTGAGGAACGAGTTTACGGGCGTCCTCACGGGCAAAAACGTCAAGTGGGGCGGGTCCTTCATCAGGCCGGAGGCCACAGGCTATGGCGCTGTCTACTTCCTGGAGGAGATGTGCAAGGACAACAACACTGTAATCAGGGGCAAGAACGTCCTCCTTTCCGGCTCCGGCAACGTCGCTCAATTTGCTTGTGAGAAGCTCCTTCAGCTCGGCGCGAAGGTCCTTACCTTTTCAGACTCCAATGGAACCATTGTCGACAAGGACGGGTTTAATGAGGAAAAGCTGGACCACCTCAAGTATCTCAAGAACGAAAAGCGTGGGCGCGTTTCTGAGTTCAAGGACAAGTATCCTGGAGTCATGTACTATGAAGGCAAGAAGCCTTGGGAGTGCTTCGAGGGCCAGGTAGATTGCATCATGCCTTGCGCCAC

>KP334147

TTCAGAGGCACGTTGGGGCTGACACTGATGTTCCTGCCGGCGACATCGGCGTCGGCGCTCGCGAGATCGGTTACTTGTACGGACAGTACAAGCGCCTGAGGAACGAGTTTACGGGAGTCCTCACGGGCAAAAACGTCAAGTGGGGCGGGTCCTTCATCAGGCCGGAGGCCACAGGCTATGGCGCTGTCTACTTCCTGGAGGAGATGTGCAAGGACAACAACACTGTAATCAGGGGCAAGAACGTCCTCCTTTCCGGCTCCGGCAACGTCGCTCAATTTGCTTGTGAGAAGCTCCTTCAGCTCGGCGCGAAGGTCCTTACCTTTTCAGACTCCAATGGAACCATTGTCGACAAGGACGGGTTTAATGAGGAAAAGCTGGACCACCTCAAGTATCTCAAGAACGAAAAGCGTGGGCGCGTTTCTGAGTTCAAGGACAAGTATCCTGGAGTCATGTACTATGAAGGCAAGAAGCCTTGGGAGTGCTTCGAGGGCCAGGTAGATTGCATCATGCCTTGCGCCAC

>KP334148

TTCAGAGGCACGTTGGGGCTGACACTGATGTTCCTGCCGGCGACATCGGCGTCGGCGCTCGCGAGATCGGTTACTTGTACGGACAGTACAAGCGTCTGAGGAACGAGTTTACGGGCGTCCTCACGGGCAAAAACGTCAAGTGGGGCGGGTCCTTCATCAGGCCGGAGGCCACAGGCTATGGCGCTGTCTACTTCCTGGAGGAGATGTGCAAGGACAACAACACTGTAATCAGGGGCAAGAACGTCCTCCTTTCCGGCTCCGGCAACGTCGCTCAATTTGCTTGTGAGAAGCTCCTTCAGCTCGGCGCGAAGGTCCTTACCTTTTCAGACTCCAATGGAACCATTGTCGACAAGGACGGGTTTAATGAGGAAAAGCTGGACCACCTCAAGTATCTCAAGAACGAAAAGCGTGGGCGCGTTTCTGAGTTCAAGGACAAGTATCCTGGAGTCATGTACTATGAAGGCAAGAAGCCTTGGGAGTGCTTCGAGGGCCAGGTAGATTGCATCATGCCTTGCGCCAC

>KP334149

TTCAGAGGCACGTTGGGGCTGACACTGATGTTCCTGCCGGCGACATCGGCGTCGGCGCTCGCGAGATCGGTTACTTGTACGGACAGTACAAGCGCCTGAGGAACGAGTTTACGGGAGTCCTCACGGGCAAAAACGTCAAGTGGGGCGGGTCCTTCATCAGGCCGGAGGCCACAGGCTATGGTGCTGTCTACTTCCTGGAGGAGATGTGCAAGGACAACAACACTGTAATCAGGGGCAAGAACGTCCTCCTTTCCGGCTCCGGCAACGTCGCTCAATTTGCTTGTGAGAAGCTCCTTCAGCTCGGCGCGAAGGTCCTTACCTTTTCAGACTCCAATGGAACCATTGTCGACAAGGACGGGTTTAATGAGGAAAAGCTGGACCACCTCAAGTATCTCAAGAACGAAAAGCGTGGGCGCGTTTCTGAGTTCAAGGACAAGTATCCTGGAGTCATGTACTATGAAGGCAAGAAGCCTTGGGAGTGCTTCGAGGGCCAGGTAGATTGCATCATGCCTTGCGCCAC

>KP334150

GATCGAGGTCCGCCGCGTCGACGACGACACGCGCGTGAAGATGATCAAGGACGCCATCGCACACCTCGACAGGCTCATCCAGACGGAGTCGAGGAAGCGCCAGGCCTCGTTCGAGGACATCCGCGAGGAGGTCAAGAAGTCTGCCGATAACATGTACCTGACAATCAAGGAAGAGATCGACACCATGGCTGCAAACTTCCGCAAGTCTCTCGCGGAAATGGGCGACACACTCAACAACGTTGAGACAAACCTCCAGAACCAGATCGCCATCCACAACGATGCCATCGCGGCCCTCAGAAAGGAGGCCCTCAAGAGCCTGAACGACCTCGAGACGGGCATCGCCACGGAGAACGCAGAGAGGAAGAAGATGTATGACCAGCTCAACGAGAAGGTCGCAGAGGGCTTTGCCCGCATCTCCGCCGCCATCGAGAAGGAGACGATCGCTCGCGAGAGGGCCGTCAGCGCCGCCACGACGGAGGCCCTCACAAACACGAAGCT

The GenBank accession numbers should appear in any publication that reports or discusses these data, as it gives the community a unique label with which they may retrieve your data from our on-line servers. You may prepare and submit your manuscript before your accessions are released in GenBank.

Submissions are not automatically deposited into GenBank after being accessioned. Each sequence record is individually examined and processed by the GenBank annotation staff to ensure that it is free of errors or problems.

You have not requested a specific release date for your sequence data.Therefore, your record(s) will be released to the public database once they are processed. If this is not what you intended, please contact us as soon as possible with the correct release date.

Since the flatfile record is a display format only and is not an editable format of the data, do not make changes directly to a flatfile. For complete information about different methods to update a sequence record, see: http://www.ncbi.nlm.nih.gov/Genbank/update.html

Any inquiries about your submission should be sent to [gb-admin@ncbi.nlm.nih.gov](mailto:gb-admin@ncbi.nlm.nih.gov)

For more information about the submission process or the available submission tools, please contact GenBank User Support at [info@ncbi.nlm.nih.gov](mailto:info@ncbi.nlm.nih.gov).

Please reply using the original subject line. This will allow for faster processing of your correspondence.

Sincerely,

Beverly Underwood

Contractor

The GenBank Direct Submission Staff

Bethesda, Maryland USA
